# Supplementary material for: Are carbon emissions trading and green financial instruments synergistic? -Comprehensive quantitative research based on content analysis
Source: PLoS One. 2024 Mar 7;19(3):e0298601. doi: 10.1371/journal.pone.0298601 (PMC10919601; doi:10.1371/journal.pone.0298601)
Supplement: S1 Data — (ZIP) [file pone.0298601.s001.zip › Data/地级市Z_F工作报告.zip!/─■╧─/╣╠╘¡╩╨/└┤╫╘╣1⁄2╓┌║┼í╛┬φ┐╦╩2╛▌═°í┐_2018.docx]

各位代表：

　　我代表市人民政府向大会报告工作，请予审议，并请市政协委员和列席人员提出意见。

　　2017年工作总结

　　去年，市人民政府认真学习贯彻党的十九大和习近平总书记来宁视察重要讲话精神，在习近平新时代中国特色社会主义思想指引下，全面落实党中央、国务院、自治区党委、政府和市委的决策部署，坚持以脱贫攻坚为统领，紧盯自治区“三大战略”，聚焦固原“六场硬仗”，继续夯实“六个基础”，推进“六大工程”，全市经济运行平稳、社会发展和谐、生态文明向好。预计完成地区生产总值270亿元，增长8%；地方一般公共预算收入16.7亿元，同口径增长20.7%；全社会固定资产投资435亿元，增长10%；社会消费品零售总额71亿元，增长9%；城乡居民人均可支配收入分别达到24700元和8500元，分别增长8%和10%。实现了新一届政府工作的良好开局。主要工作成效是：

　　（一）提升了精准脱贫质量。坚持一手抓脱贫销号，一手抓巩固提升，销号贫困村200个、减贫9.4万人，贫困发生率下降到8%。2016年小康实现程度72.25%。脱贫措施更实。开展“五个回头看”，精准识别贫困人口18.8万人。培育“有土”富民产业，实施“5·30”及其倍增计划，6.2万建档立卡户从事种植和养殖业；扶持“离土”创业增收，培训农村劳动力5.4万人，劳务收入支撑建档立卡户收入增长13%。对标补齐短板，新修村道3044公里，通路率95%；完成15项农村饮水安全工程，通水新增2.3万户，自来水入户率94.5%；改造危房危窑1.93万户，完成140.2%；3.24万人搬进了温暖的新家。金融扶贫更实。放大“产业+扶贫+财政+金融”模式，将担保基金和风险补偿基金从12.5亿元提高到18.3亿元，为7.9万贫困户贷款33.8亿元，户均4.3万元，覆盖率80.1%，“扶贫保”实现贫困人口全覆盖。“蔡川模式”在中央电视台新闻联播报道。脱贫机制更实。建立市级领导带队明察暗访等机制，开展整治脱贫工作作风不实暨扶贫领域专项资金监督检查行动，完成扶贫领域120个问题整改；建立“有进有出”的动态管理机制，清退10326人，补录12086人；以正向激励促更多的贫困户主动脱贫。建立5亿元担保基金，已放大担保贷款21.1亿元，扶持农村“两个带头人”增强带动能力，这一做法得到中央领导充分肯定，全国、全区经验交流现场会在固原召开，中央、自治区媒体进行了集中报道，反响很好。

　　（二）提升了产业发展质量。深化供给侧结构性改革，推动五河流域产业带建设。破局产业升级。谋划争取支持的精准扶贫、产业发展等项目172个总投资1520亿元，争取国家、自治区各类资金104亿元；与工商银行、建设银行等签订了400亿元信贷协议，国投、国开行授信市扶贫开发投融资公司10亿元，年末金融机构存贷款余额分别达到560亿元和458亿元，贷存比81.8%，较年初提高12个百分点；与海升集团、汇源果汁、金田阳光等行业龙头合作取得实质性进展，全市招商引资到位资金203亿元，增长20%。农业调优提质。积极应对春季旱情，粮食生产平稳，总产78万吨。固原国家农业科技园区通过科技部验收。新建肉牛养殖园区10个、示范村20个，肉牛饲养量达到111万头，增长2.8%，其中安格斯纯种基础母牛4万头。推广马铃薯主食化品种2万亩；新发展冷凉蔬菜2.8万亩。创建杂粮、油料标准化生产基地20个。发展红梅杏、枸杞等特色经济林6.6万亩，养殖中蜂5.2万群。农牧业综合机械化率58.7%，处于全国丘陵山区领先水平。固原马铃薯、胡麻油、黄牛、彭阳红梅杏、朝那鸡5个产品获国家地理标志认证，“五谷六盘”杂粮等13个产品获宁夏名牌。预计农业增加值增长4.3%。工业增添动能。培育“专精特新”企业22家、示范企业14家。王洼煤矿600万吨改扩建项目基本完工，金昱元盐化工一期建成试车，融信华创西吉风力发电项目一期并网发电，六盘山绿色中药材资源开发及交易市场、山东红帆智能供变电系统和云轨支架项目落地，宁夏丰源20万锭纺纱项目开工建设，填补了我市纺织产业空白。预计工业增加值增长11%。服务业欣欣向荣。以创建自治区全域旅游示范市为目标，加快14条旅游专线及综合配套服务设施建设，完成茹河瀑布等景区改造提升。大店、龙王坝、新和、新月、阳洼等13个旅游示范村特色初显，乡村游、生态游持续升温，全年接待游客490万人次、旅游社会收入29.1亿元，分别增长18%和19%。固原市荣获最美中国“全域旅游创建典范城市”，泾源县荣获全国十佳生态旅游城市。培育电商扶贫示范村5个、龙头企业5家、经销企业30家，京东—固原扶贫馆开馆运营，农产品线上销售突破1亿元；设立特色农产品外埠窗口2个。餐饮住宿业发展良好。预计服务业增加值增长8%。

　　（三）提升了城乡环境质量。坚持城乡统筹、建管并举，不断优化人居环境，国家新型城镇化试点工作有序推进，常住人口城镇化率提高3个百分点，达到49%。城乡面貌变化喜人。把市区旧城改造与海绵城市建设有机结合、整体推进，博物馆等9个片区200万平方米棚改全部开工建设，清水河两岸等150万平方米棚改拆迁工作进展顺利；海绵化改造小区90个323万平方米，建成古城墙遗址公园一期。进一步理顺城市管理体制，开启环境卫生“以克论净·深度保洁”模式，成功创建国家园林城市和自治区文明城市。泾源20公里旅游服务带和隆德渝河、彭阳茹河综合治理成为固原的新亮点。集中实施了张易、将台、观庄、兴盛、城阳等10个美丽小城镇和三十里铺、西坪、新庄、杨岭、高庄等55个美丽村庄。习近平总书记视察过的将台、杨岭和姚磨发生喜人变化，实现了百姓安居、环境改善、城乡变美。基础设施日趋完善。实施了一批交通、水利、信息化等城乡基础设施项目，支撑经济社会发展。李克强总理关心的三条对外通道S25线泾河源至华亭、S60线西吉至会宁、G327线沟圈至彭阳全面开建，新建农村公路522公里，开通固原—福州航线。固原市入选全国第一批公交都市建设城市，原州区获全国首批“四好农村路”示范县，西吉县入选全国城乡交通运输一体化示范县。宁夏中南部城乡饮水安全工程项目区全部通水，泾河源镇等7处抗旱应急水源工程建成投用，新建李渠等水库6座、改造提升寺口子等水库6座，竭力解决了隆德县城应急饮水困难。新增高效节水灌溉面积5万亩，累计达到61.8万亩。新建4G基站1092座，行政村光纤到户覆盖率100%。生态环境持续改善。建立市域内生态补偿机制；实施六盘山重点生态功能区400毫米降水线以上区域造林绿化工程，完成新一轮退耕还林5.3万亩，共造林67.6万亩，森林覆盖率提高2.3个百分点，达到25.1%。治理水土流失面积320平方公里。清水河国家湿地公园通过国家林业局验收。开展蓝天、碧水、净土和“绿盾2017”专项行动，依法关停河道采矿厂、洗砂厂、砖厂等136处，淘汰万吨以下马铃薯淀粉加工企业34家；拆除自然保护区违规建筑81万平方米，恢复植被199万平方米，完成了六盘山国际旅游度假区项目生态环境综合整治。全面推行河长制，从源头、河道、流域系统治理五河，市县城区污水处理厂改造提标和工业园区污水处理厂正式投运，三营、神林等6个乡镇污水处理厂开建，泾河水质稳定在Ⅱ类，葫芦河、渝河、茹河稳定在Ⅳ类以上，渝河治理模式被环保部总结为“渝河经验”。铁腕防治扬尘、烟尘，淘汰20蒸吨以下燃煤锅炉132台、黄标车1568辆，影响空气质量的主要污染物PM10进一步下降，优良天数达到90%以上，稳居全区第一。

　　（四）提升了民生保障质量。认真办理自治区民生计划，市政府为民承诺的13件实事全部完成。教育政策顺应民心。在全区率先出台教育填平补齐政策，实现农村学生各个学段全程资助。新建改建幼儿园147所，改造薄弱学校234所，原州区、隆德县、泾源县、彭阳县义务教育均衡发展通过国家评估认定，西吉县通过自治区验收。实施“名师、名校、名校长”培育工程，创新推出教师职称评定“地方粮票”，命名首批“六盘名师”，尊师重教氛围更浓，广大教师的育人热情充分迸发。医疗改革化解民忧。出台提高农村居民医疗保障水平和建立重特大疾病补充医疗保险政策，农村居民年度内住院医疗费用个人负担比例由23.5%控制到10%以内、建档立卡贫困户自付费用累计不超过5000元，重特大疾病年度最高救助限额由8万元提高到16万元。综合医改和城市公立医院改革稳步推进，市医院迁址运行良好，综合医院上等级创建活动有序推进；实施医疗人才“双优”工程，建立京宁、闽宁、沪宁合作机制和县乡医联体39个，基层诊疗量从83%提高到96%，逐步扭转群众“不管大小病都跑大医院”的传统就医格局。群众文化异彩纷呈。建成农村综合文化服务中心806个，实现村级全覆盖，创建市级文化大院25个，入围创建国家公共文化服务体系示范区，文化大院创建经验在西部五省区推广。创排了《红旗漫卷六盘山》《山桃花开》等剧目。成功举办第四届宁夏六盘山登山节和第二届全市农民运动会。社会保障温暖百姓。建成5个创业孵化园、5条创业示范街，发放创业担保贷款10.3亿元，创业带动就业9549人，新增城镇就业10045人；转移就业31.97万人，创收60亿元。“五险合一”经办体制改革基本完成，实现了与全国31个省份7443家医疗机构联网结算。推进农村低保制度与扶贫开发政策有效衔接，提高贫困人口保障覆盖面。市养老综合服务中心主体竣工，建成老年活动中心5个、农村敬老院7个、老饭桌20个。社会治理守护安宁。推进“七五”普法和法治政府建设，在全区率先打造“一村一法律顾问”援助品牌；扎实开展信访维稳、社会治安、安全生产大排查、大调处、大管控，全年没有发生影响社会安定的重大案（事）件和较大以上生产安全事故，为党的十九大胜利召开营造了良好的社会环境。成功破获特大电信网络诈骗案，受到公安部和自治区党委、政府充分肯定。原州区被评为“全国平安建设先进县”。精神文明创建成效明显，头营镇圆德村等10个单位入选第五届全国文明单位，宁夏师范学院入选第一届全国文明校园；王黎君荣获第六届全国道德模范提名奖。加强民风建设，所有行政村建立“一约四会”制度，把“软约束”变成“硬杠杠”，移风易俗取得新成绩。启动“拥护核心感党恩、同心携手奔小康”民族团结进步创建主题活动，原州区、彭阳县被命名为全国民族团结进步创建示范区；坚持宗教中国化方向，依法加强宗教事务管理和专项治理，民族团结、宗教和顺、社会稳定的良好局面不断巩固。

　　（五）提升了重点改革质量。从经济发展重点、社会关注焦点、群众反映难点入手，推动重点改革取得阶段性成效。“放管服”改革惠企利民。市本级集中行政审批事项从145项压减到123项，办理时限压缩78%，非行政许可事项全部“清零”。全区政务服务“一张网”固原站上线运行，60%的事项实现不见面办理。市本级建设项目“多评合一”“多图联审”改革模式走在全区前列。全面实行“多证合一、一照一码”登记制度改革，实现了企业注册登记“三十三证合一”，新登记各类市场主体6640户，同比增长23.4%。“空间规划”改革圆满交卷。划定城镇、农业、生态空间及城镇开发边界、永久基本农田、生态保护红线；编制了市区和泾源县空间规划，西吉县、隆德县、彭阳县空间规划编制工作有序推进，初步形成了全市一本规划、一张蓝图，为科学指导城乡建设、产业发展、生态保护提供了基本遵循。农村综合改革破冰前行。建成固原三农工作站和5个县级农村产权流转交易中心，农村集体资产股份权能（投改股）改革试点顺利推进，启动农村宅基地“房地一体”不动产确权登记颁证。完成市县（区）供销社机关事业单位法人登记，改造基层社10个，建成农业社会化综合服务站15个。完成小型水利工程管理体制改革，彭阳“互联网+水利”管理模式在全区推广。

　　（六）提升了政府服务质量。坚定政治立场。市政府把学习宣传贯彻党的十九大精神作为政治必修课和工作主题，深学践悟。坚持把政治纪律和政治规矩挺在前面，增强“四个意识”，坚定“四个自信”，坚决落实自治区党委、政府和市委各项决策部署；加强政府党组建设，修订了工作规则，坚持重大问题集体研究，重大事项向市委请示。聚焦工作落实。积极开展“振奋精神、实干兴宁”大讨论活动，按照“生态优先、富民为本、绿色发展”的定位要求和市委的决策部署，出台了贯彻自治区“三大战略”，建设西部地区绿色发展示范市、全国脱贫攻坚示范市、全国生态文明示范市三个实施方案，成为今后工作的总抓手；严格落实领导干部责任制，实行重点工作“四个清单”管理制度。办结区、市人大代表议案建议28件、政协委员提案140件，提请市人大常委会审议通过《固原市须弥山石窟保护条例》。从严廉洁行政。自觉执行党风廉政建设责任制，全面推广涉农惠农项目资金“331”监管模式，开通“12345”便民服务热线，加大行政监察、审计监督和整治“四风”力度，全年共立案查处各类违纪违法案件341件，给予党纪政纪处分341人。严格控制政府债务结构和风险。统计、人防、地震、气象、档案、地方志、残疾人、工会、共青团、妇女儿童等工作取得新的进步。

　　各位代表，过去的一年，政府各项工作取得喜人成绩。这得益于自治区党委、政府和市委的正确领导、充分信任，得益于市人大、市政协的有效监督、大力支持，得益于全市广大干部群众的同心协力、务实苦干。在此，我代表市人民政府，向全市各族人民，向人大代表、政协委员、各民主党派、工商联、无党派人士和各人民团体，向中央、自治区驻固单位、人民解放军、武警官兵及公安干警，向关心支持固原改革发展的社会各界，表示崇高的敬意和衷心的感谢!

　　总结过去，发展不充分仍然是固原最大的实际，脱贫小康的路上还面临着许多困难和问题。一是脱贫帮扶不实，内生动力不足。脱贫攻坚的责任没有完全到村到户，派驻村干部和帮扶到户到人措施不精准，贫困户增收渠道单一；部分群众习惯凡事等政策、靠帮扶、要支持，“我要脱贫”的内生动力不足。二是城市经济滞后，居民增收乏力。产城融合度低，产业支撑增收能力弱，顺应潮流的生态经济、数字经济、共享经济等产业缺企业引领、人才支撑，电子商务、咨询服务等新业态发展缓慢，城镇居民增收的来源较少。三是富民产业不强，升级步伐缓慢。特色农业发展仍处于种养的初级阶段，缺乏龙头企业带动，产业链条短；新品种、大品牌培育不足，市场竞争力不强；新兴产业类项目储备少，产业类投资支撑区域发展、可持续发展后劲不足。四是环保欠账较多，治理任务艰巨。五条河流均为季节性河流，存在自净能力差和治污设施滞后的双重矛盾，五河水质稳定达标排放的难度大、成本高；城区散户居民多，散煤用量大，粉尘、扬尘、烟尘并存，防治任务重；自然保护区落界不清，管理体制不顺的问题未根本解决。五是创新担当不佳，求真务实不够。一些干部思想观念守旧，创新意识欠缺，习惯于传统思维方式、速度情结和发展路径；部分干部责任意识、担当意识不强，抓落实多“击鼓传花”、少“精益求精”；会多文多、调研少，不会为、乱作为的不良作风未根本改变。这些问题，我们必须痛下决心，坚决改进！

　　2018年重点工作

　　今年，是我市决胜脱贫攻坚的关键一年。我们要坚决破除传统思维定势，树立问题目标导向，坚持一张蓝图绘到底，闯出一条山绿与民富双赢、脱贫与小康共进的新路子。

　　按照市委四届三次全会部署，政府工作的总体要求是：以习近平新时代中国特色社会主义思想为指引，深入学习贯彻党的十九大精神，牢固树立和践行新发展理念，全面落实自治区第十二次党代会和市委四届二次、三次全会精神，围绕经济繁荣、民族团结、环境优美、人民富裕目标，把握生态优先、富民为本、绿色发展定位，深入实施“三大战略”，认真落实“五个扎实推进”，坚持以脱贫攻坚统领经济社会发展全局，坚持稳中求进工作总基调，坚持高质量发展根本要求，全力打好“六场硬仗”，加快“四个示范市”建设，维护社会和谐稳定，推进全面从严治党，振奋精神，实干兴宁，决战脱贫攻坚、决胜全面小康，以优异成绩向改革开放40周年和自治区成立60周年献礼。

　　主要目标是：地区生产总值增长7.5%，全社会固定资产投资增长9%，社会消费品零售总额增长9%，地方一般公共预算收入和支出分别增长8%和9%，城乡居民人均可支配收入分别增长8%和9%，完成自治区下达的各项约束性指标。重点抓好以下工作：

　　（一）聚焦隆泾彭三县摘帽，打好脱贫富民决胜战。全面落实我市贯彻自治区脱贫富民战略建设全国脱贫攻坚示范市“35条”，统筹脱贫攻坚和区域发展，做好“准”和“实”的固原脱贫文章，销号贫困村58个、减贫4万人。对标发力扶真贫。整合资金和政策，聚焦74个深度贫困村，全面实现“两不愁、三保障”。坚持不懈抓产业扶贫、就业扶贫，瞄准特定贫困群众精准帮扶。把闽宁协作、“百企帮百村”等社会帮扶向深度贫困乡村倾斜，集中力量啃下硬骨头。对539个脱贫村实施基础设施、公共服务提升工程，全面完成1.1万户危房改造和2900户1.2万人易地扶贫搬迁，从实从细补齐脱贫短板和任务，成效真实。紧扣增收真扶贫。实施金融扶贫提质工程，建成100个金融扶贫示范村，新增各项贷款90亿元，争取贫困户户均贷款达到5万元、脱贫户6—10万元。落实“三大三强”行动，提升“两个带头人”工程，推广原州区姚磨跨村跨乡跨区域带动模式，把党支部建在产业链上，让老百姓富在产业链上。“有土”扶贫重点实施好“5·30”倍增计划，扶持特色种养业标准化和高质量发展，培育新型经营主体430个，完善利益联结和产销对接组织模式，实现户均有1—2个致富产业。“离土”扶贫对标职业市场，定向培训3.6万人，订单培训保姆、厨师、护工等5000人；多渠道搭建“扶贫车间”，打造自治区级创业示范基地2个，培育创业孵化基地5个，扶持新增创业创新主体2000户，创业带动就业8000人；转移就业30万人以上，创收65亿元以上，贫困户经营性收入、工资性收入增长10%以上。正向激励扶志气。脱贫先壮志，丰富脑袋才能装满口袋。调整政策扶持环节，采取贴息奖补、项目奖励等措施，激励贫困村、贫困户变“等着扶”为“干着扶”，大张旗鼓地表彰先进；通过开展农民培训教育年活动，让农民知晓党的“三农”政策，增强致富技能，推进移风易俗，进一步激发贫困群众摆脱贫困的斗志和奔向幸福美好生活的动力。精准退出脱真贫。紧盯国家对贫困县摘帽的评估指标，坚持缺什么、补什么，干部下足苦功夫，换得百姓乐开怀。认真开展扶贫领域“作风建设年”活动，及时纠正各类问题，实现贫困人口信息化管理、扶贫资源阳光化使用、贫困农户精准化扶持、脱贫产业稳定化增收；靠实市县乡抓落实的工作责任，完成隆德、泾源、彭阳摘掉贫困县帽子的历史壮举！

　　（二）聚焦产业延长链条，打好创新驱动攻坚战。全面落实我市贯彻自治区创新驱动战略建设西部地区绿色发展示范市“30条”，按照高质量发展要求，加快构建现代化产业体系。科技创新引领发展。推进“121”工程，以企业为主体，落实科技创新奖励政策，支持引导产学研协同创新，促进科技成果转移转化。整合政府扶持各产业创新资金，引导撬动企业和银行加大研发投入，实现R&D投入强度翻番。培育国家高新技术企业2家、自治区科技型企业10家。提升固原国家农业科技园核心区功能，加快建设四县现代农业科技示范园区，推广应用新品种、新技术，提升良种化、标准化和集约化水平。落实人才改革意见，建立固原人才智库，让创新和科技人才成为高质量发展的制胜法宝。特色农业“接二连三”。要“反弹琵琶”，坚持质量兴农、绿色兴农，突出抓好五县区“5个10亿农业产业集群培育”行动。实施汇源综合扶贫开发园、山东水发马铃薯循环产业园、宁夏森沃花卉及冷凉蔬菜现代农业科技园、重庆恒都10万头肉牛精深加工等项目，延长农产品价值链。每个县区至少引进2家产值过亿元的农产品精深加工企业。加强与宁夏宁苗、北京克劳沃、陕西海升等集团合作，把“一棵树、一枝花、一棵草、一株苗”培育成固原山绿与民富的种子，种出风景，种出财富。依托京东—固原扶贫馆，对接加盟全国知名市场，完善农产品质量安全追溯体系，实现小产业连上大市场，六盘山农产品卖出好价格。工业延链育新。坚持生态、环保、节水优先原则，实施“百家规上企业突破”和“非公经济提升”计划，引导现有企业“联强靠大”，延伸产业链，对标大市场，实现产业信息融合发展；依据市场有需求、企业有盈利、固原有优势，引进扶持培育创新型、创业型、劳动密集型企业，创造新时代固原新产业。开工建设一批产业新项目，建成投产宁夏丰源20万锭纺纱项目。出台工业园区转型发展实施意见，低成本化改造工业园区，形成“一园一业一特色”。年内新增规上工业企业15家。全域旅游达标升级。全面落实自治区全域旅游“26条”，完善旅游规划，实施旅游“双融”行动，创建六盘山长征5A级景区，改造提升将台堡红色教育基地，建成宁夏娅豪兴盛滑雪场和14条218公里旅游专线。扶持开发乡村民俗体验等增加老百姓收入的“短平快”项目，打造大店、冶家村等旅游示范村13个、特色农家乐200户、智慧民宿100家。办好六盘山山花节等系列节会，充分挖掘地域文化内涵，讲好固原故事，推动旅游和文化深度融合。发挥“旅游+”的综合带动作用，开发避暑游、康养游、乡村游、冬季游等产品，加大中高端旅游产品供给，力争全年接待游客550万人次以上，旅游社会收入40亿元以上，使“天高云淡六盘山”名扬四海、享誉全国。服务业提质培强。制定现代物流业发展规划，开工建设金田阳光义乌小商品城，建成固原市公益性农产品批发市场、派胜世贸金街；实施“消费回流”计划，出台消费回流政策，支持打造精品街区、特色夜市，吸引外流消费回归、周边消费流入。抓好5县区电商产业园建设，培育电商企业10家、京东便利店100家；建成农产品冷链物流体系和3个“菜蓝子”连锁超市，进一步做活城乡消费市场。培育发展信息技术、教育培训、养老服务、会展博览、住房租赁等新业态。引进金融机构2家以上，提升金融支持壮大实体经济能力。

　　（三）聚焦五河水质提标，打好生态环境保卫战。全面落实我市贯彻自治区生态立区战略建设全国生态文明示范市“30条”，抓好生态修复、河道治理、控源截污，守护好固原的绿水青山。植绿固原大地。推进造林绿化工程，实施六盘山三河源水质涵养、湿地保护与修复、市区生态屏障和绿色廊道、生态监测及人工增雨等项目，造林65万亩，森林覆盖率提高到26%以上。治理蒋口等小流域300平方公里。守卫碧水蓝天。突出全民共治、源头防治，坚决完成中央环保督察反馈问题整改，坚决制止和惩处破坏生态环境行为。对标“大气10条”，推进“蓝天行动”，完成市区1000多家餐饮单位清洁能源改造；加快固原天楹循环产业园项目建设，实现秸秆有效利用和垃圾无害化处理，严控烟尘、扬尘等新增排放，市区PM2.5、PM10稳中有降，确保空气质量优良天数稳定在90%以上。对标“水10条”，推进“碧水行动”，压实河长责任，严格河湖岸线管控和水源地保护，加快五河流域水环境综合治理；建成城市污水处理厂污泥规范化处理、城镇排污管网和人工湿地项目，确保污水达标排放，坚决消除清水河劣五类水质，实现五河流域水质达标并稳定提升。厚植绿色根基。对标“土10条”，推进“净土行动”，减少农药、化肥使用，大幅增加有机农家肥、有机农药使用量，严控农村面源污染，农田残膜回收率达到90%以上。开展全市生态环境现状调查，完成城镇开发、永久基本农田、生态保护红线落界工作。严格产业项目准入，加快工业固体废弃物综合利用，从源头上为生态环境减负。落实最严格的水资源管理制度，市区创建自治区水生态文明城市，四个县争创国家节水型城市。

　　（四）聚焦乡村振兴战略，打好城乡统筹大会战。坚持城乡一体、产城融合思路，持续改善城乡人居环境。提升中心城市品质。按照固原市空间规划，修编城市总体规划，完善控制性详规和综合交通、夜景照明、城市双修等系列规划。完成市区旧城（棚户区）改造5000户，实施35个老旧小区海绵化改造，建设城市公园5个，拓展城市休闲空间，不断满足人民日益增长的优美生态环境需要。深化城市“以克论净·深度保洁”机制，改进社区物业服务管理，巩固“五城联创”成果，启动全国文明城市、双拥模范城市和国家卫生城市、森林城市等“九城同创”工作。实施乡村振兴战略。围绕实现农业强、农村美、农民富目标，建立完善城乡融合发展体制，做好乡村振兴与脱贫攻坚的政策衔接、机制整合和工作统筹，推动农村基础设施、公共服务提档升级。打造张易、马建、陈靳、黄花、孟塬等7个特色小镇，沿旅游环线集中建设双泉、三合、锦华、集美、白岔等51个美丽村庄。开展“百村示范、千村整治”工程，完成524个行政村人居环境综合整治，建制镇和中心村生活污水处理率达到50%以上，农村自来水普及率达到95%以上；启动农村“厕所革命”，旅游乡镇、旅游村卫生厕所全覆盖，努力补齐影响群众生活品质的短板。夯实发展基础。全力以赴争取宝中快铁、G85线南坪至高寨塬高速公路项目开工，加快三条对外通道建设，新（改）建国省干线11条369公里和四好农村公路400公里；实施六盘山机场扩建停机坪工程；推进公交都市示范创建工作，优化市区路网，改建城市进出快速通道，解决交通拥堵问题。新建、改造提升南城拐子、什字等水库11座，新增高效节水灌溉面积4万亩。实施新一轮农村电网改造升级工程，完善天然气管网和配套基础设施。启动“智慧固原”建设，促进互联网+扶贫监测、城市管理、健康医疗、文化旅游等融合应用，让固原搭上新时代的信息快车。

　　（五）聚焦公共服务提升，打好民生改善持久战。办好自治区民生计划和我市16件民生实事（见附件2），不断满足人民美好生活需要。优先发展教育事业。改善290所中小学办学条件，新（改）建幼儿园122所，完成固原一中初中部、固原二中扩建、民族职业技术学院提升改造和市幼儿园迁建等项目；支持和规范社会力量办学，有效缓解教育资源分布不均衡和“上好学难”“大班额”问题。完善职业教育体系，深化产教融合、校企合作工作。开展教师素质、教育质量“双提升”行动，推进与苏州市、深圳市的名校联合办学，提高教育教学质量。推进健康固原建设。持续深化综合医改，完善公立医院运行机制和分级诊疗就医体系，提升市县综合医院等级创建水平，与北京宣武医院、北大第一医院等合作组建医联体，改扩建市中医院和市妇幼保健中心，建成社区卫生服务中心2个；开展医师素质、医疗质量“双提升”行动，持续解决好“看病难”“看病贵”问题。发展健康养老产业。深入开展爱国卫生运动，预防控制重大疾病；加强食品安全监管，让人民吃得放心。实施文化惠民工程。推进全国公共文化服务体系示范区建设，打造市级示范性文化大院20家、县级50家；建成原州区、彭阳县2个全民健身中心，开工建设市体育场和隆德县文化馆。打磨提升《红旗漫卷六盘山》，举办首届文学艺术节，让群众享受更加美好的文体生活。提升平安固原水平。全面推行“网上信访”模式，妥善解决群众利益诉求。加快“雪亮工程”建设，完善立体化治安防控体系，依法严厉打击黄赌毒黑拐骗等违法犯罪活动，使人民群众的安全感更可持续。严厉打击非法集资、金融诈骗行为。全面落实安全生产责任，强化重点行业领域安全生产监管，坚决遏制重特大安全事故。以争创全国民族团结进步示范市为抓手，深化民族团结进步教育，使“三个离不开”“五个认同”思想更加深入人心，促进各民族像石榴籽一样紧紧抱在一起，提升“六盘儿女一家亲”品牌的影响力、感召力；依法加强宗教事务管理，坚决防范和治理“回族字眼概念泛化”“阿化”“沙化”等苗头性、倾向性问题，促进宗教和顺平静。支持工会、共青团、妇联、残联、科协、文联等群团组织更好地开展工作。

　　（六）聚焦发展机制创新，打好改革开放突围战。坚持改革不停顿、开放不止步，为经济社会高质量发展注入强劲动力和不竭活力。用改革的方法破解难题。针对环境不优、服务滞后的问题，持续深化“放管服”改革，推广“互联网+政务”，实现80%的政务服务事项不见面办理；推行“证照分离”改革，优化服务企业环境，做好“店小二”式服务，构建亲清新型政商关系，全面推行“双随机、一公开”监管。完成新一轮政府机构改革，组建市法制办、金融工作局。针对村集体收入少、农民财产收益低的问题，开展农村土地“三权分置”加监督权的“四权分置”试点，有序推进农村土地承包经营权、农民住房财产权抵押贷款，全面推开农村集体资产股份权能（投改股）改革，增加农民财产收益。针对国有资产增值收益低、主业不清不强的问题，明晰九龙、旅游、交通、扶贫、经济技术开发区五家国有公司的发展方向、重点，强壮自我发展、抵御风险的筋骨；规范政府举债行为，制定化解债务风险方案；优化财政支出结构，统筹安排市本级预算资金29亿元，重点保障实施好“三大战略”、打好“六场硬仗”，提高资金使用效率。以开放的姿态迈向小康。主动融入国家“一带一路”倡议，积极参与宁夏内陆开放型经济试验区建设，有效承接长三角、珠三角、京津冀等发达地区产业转移和创新成果转化，务实推进与陕甘宁蒙毗邻城市合作；进一步提升闽宁协作质量和水平，争取外省市在固原建设“共享工业园”。继续开展招商引资年活动，以商招商、以诚招商，全年招商引资到位资金230亿元以上，增长15%以上，产业类项目达到60%以上。

　　各位代表，新时代要有新气象、更要新作为。我们必须围绕建设人民满意的服务型政府，旗帜鲜明讲政治，树牢“四个意识”，坚定“四个自信”，做到“四个看齐”；坚决维护以习近平同志为核心的党中央权威和集中统一领导，忠诚核心、拥护核心、爱戴核心；坚持用习近平新时代中国特色社会主义思想武装头脑、指导实践、推动工作。

　　一是把政治定力作为首要标准，笃行心中有党。加强政治理论、专业知识学习，提高政治站位、丰富知识素养，不断增强干部的战略思维、创新思维、辩证思维、法治思维、底线思维，切实提高高层次谋划、高质量发展、高水平落实的能力。扎实开展“不忘初心、牢记使命”主题教育和干部素质提升年活动，坚定理想信念，锤炼党性修养，永葆忠于党、忠于事业、忠于人民的政治本色，在政治立场、政治方向、政治原则、政治道路上同党中央保持高度一致，坚决执行党的理论、路线、政策，坚决贯彻党中央、国务院、自治区党委、政府和市委的决策部署，切实提升政治执行力。加强市政府党组工作，自觉贯彻执行民主集中制等各项制度，把党领导一切贯穿于政府工作全过程，对市委部署的工作坚决执行、立即落实、确保质量；坚持重大事项向市委请示、向人大报告、向政协通报，不越权办事、不先斩后奏。

　　二是把人民需求作为第一选择，笃行心中有民。造福人民是我们最大的政绩。自觉践行以人民为中心的发展思想，树立群众观点，坚持群众路线，大兴调查研究之风，想问题、作决策、抓工作坚持从群众中来、到群众中去，集思广益、汇聚民智，真正做到决策部署依民而定、办法措施为民而出。牢固树立正确政绩观，真正静下心来多做打基础、利长远、惠民生的工作，在创业就业、产业增收、公共服务等方面下功夫、补短板，让人民群众能真正感受到发展的“温度”。

　　三是把务实高效作为行政追求，笃行心中有责。坚守献身党和人民事业的崇高情怀，着眼解决问题下好每一盘棋、走好每一步路、干好每一件事，向人民群众交上一份满意的答卷。扎实开展干部作风转变年活动，出台落实重大决策部署的暂行办法，在打好“六场硬仗”和建设“四个示范市”中实行一项任务、一名责任领导、一套推进方案、一个督查考核奖惩办法的“四个一”工作落实机制。完善任务、项目、责任清单制度，实施好“三大战略”“六场硬仗”118个重点项目（见附件1）。坚决向庸懒散、不作为、慢作为、乱作为等问题亮剑。按照抓重点、补短板、强弱项的要求，主动“沉下去”服务企业、服务群众，积极“走出去”招项目、引资金，全年争取国家、自治区各类项目资金120亿元，带动民间投资200亿元以上。

　　四是把守法廉洁作为立身之本，笃行心中有戒。坚持把纪律和规矩挺在前面，做到按规矩、按程序依法办事。政府系统各级干部带头尊法学法守法用法，运用法治思维和法治方式深化改革、推动发展、化解矛盾、维护稳定；自觉接受人大依法监督、政协民主监督以及监察监督、司法监督、社会监督。支持监察委、法院、检察院依法行使职权。严格落实中央八项规定实施细则和区、市党委有关要求，把践行“三严三实”贯穿于全部工作生活中，切实防范和纠正形式主义、官僚主义等“四风”隐性、变异问题，不越法律“底线”，不碰纪律“红线”，做到勤勤恳恳为民、兢兢业业干事、清清白白做人。严格履行市政府党组全面从严治党主体责任，认真落实“一岗双责”，深入推进政府系统党风廉政建设；严控“三公”经费，压缩一般性支出；加强政府投资类项目和资金监管审计，开展扶贫领域腐败和作风问题专项治理，营造清正、清廉、清明的良好政风。

　　各位代表，今年是自治区成立60周年。我们要高质量办好人民期盼、政府所能的实事好事，高水平办好展览展示、特色节会、文艺汇演等系列庆祝活动，努力增进百姓福祉，充分展示固原发展成就。

　　各位代表，天道酬勤，幸福都是奋斗出来的。让我们更加紧密地团结在以习近平同志为核心的党中央周围，高举中国特色社会主义伟大旗帜，在自治区党委、政府和市委的坚强领导下，不忘初心、牢记使命，振奋精神、实干兴宁，为实现经济繁荣、民族团结、环境优美、人民富裕，打赢脱贫攻坚战、决胜全面小康，奋力谱写新时代固原发展新篇章而努力奋斗!
